# Factors associated with long-term survival in gemcitabine-concurrent proton radiotherapy for non-metastatic locally advanced pancreatic cancer: a single-center retrospective study

**DOI:** 10.1186/s13014-022-02001-w

**Published:** 2022-02-10

**Authors:** Yuta Ogura, Kazuki Terashima, Yoshihide Nanno, SungChul Park, Masaki Suga, Daiki Takahashi, Yoshiro Matsuo, Nor Shazrina Sulaiman, Sunao Tokumaru, Tomoaki Okimoto, Hirochika Toyama, Takumi Fukumoto

**Affiliations:** 1 Division of Hepato-Biliary-Pancreatic Surgery, Department of Surgery, Kobe University Graduate School of Medicine, 7-5-2 Kusunoki-cho, Chuo-ku, Kobe, Hyogo 650-0017 Japan; 2 Department of Radiology, Hyogo Ion Beam Medical Center, 1-2-1 Kouto, Shingu-cho, Tatsuno, Hyogo 679-5165 Japan; 3 Department of Radiation Physics, Hyogo Ion Bseam Medical Center, 1-2-1 Kouto, Shingu-cho, Tatsuno, Hyogo 679-5165 Japan

**Keywords:** Pancreatic cancer, Proton radiotherapy, Gemcitabine, Chemoradiotherapy, Retrospective study, Prognostic factor, Long-term survival outcome

## Abstract

**Background:**

Factors associated with long-term survival in gemcitabine-concurrent proton radiotherapy (GPT) for non-metastatic, locally advanced pancreatic cancer (LAPC) remain unclear. This study aimed to determine the factors associated with long-term survival in GPT for non-metastatic LAPC.

**Methods:**

The medical records of 123 patients with LAPC treated with GPT between February 2009 and December 2019 at Hyogo Ion Beam Medical Center were retrospectively reviewed to assess the factors associated with long-term survival outcomes.

**Results:**

The median overall survival of the total cohort treated with GPT was 18.7 months. The 1- and 2-year overall, local progression-free, and progression-free survival rates were 70.4% and 35.7%, 78.2% and 59.0%, and 38.6% and 20.8%, respectively. Multivariate analysis revealed that LAPCs at the pancreatic body-tail and those without anterior peripancreatic invasion were independently associated with longer overall survival (*P* = 0.040 and *P* = 0.015, respectively). The median overall survival of patients with LAPC at the pancreatic body-tail and those with LAPC without anterior peripancreatic invasion were 24.1 and 28.1 months, respectively. LAPCs at the pancreatic body-tail had a higher volume ratio irradiated over 60 Gy equivalents at gross tumor volume than those at the pancreatic head (*P* < 0.001). LAPCs with anterior peripancreatic invasion had more peritoneal recurrence within 6 months after GTP than those without anterior peripancreatic invasion (*P* = 0.039).

**Conclusions:**

GPT is a promising treatment option for patients with LAPC at the pancreatic body-tail and those with LAPC without anterior peripancreatic invasion.

## Background

Pancreatic cancer has a poor prognosis and is the fourth leading cause of cancer-related deaths in Western countries [1, 2]. Although radical surgical resection is the only potentially curative treatment, more than 30% of patients are diagnosed with unresectable pancreatic cancer due to extensive vascular involvement without distant metastasis (locally advanced pancreatic cancer [LAPC]) [3]. The standard treatment for LAPC is chemotherapy or chemoradiotherapy [4], and recent studies have shown that intensive chemotherapies, such as multiagent 5-fluorouracil, leucovorin, irinotecan, and oxaliplatin (FOLFIRINOX) and gemcitabine plus nab-paclitaxel (GnP), achieved long-term overall survival (OS) [5, 6]. Other studies have reported that the combination of radiotherapy with chemotherapy improves local control and prolongs OS compared with chemotherapy alone [7, 8]. Since approximately 30–40% of patients with LAPC die of local progression without developing distant metastases [9, 10], chemoradiotherapy with high local tumor control could be a feasible treatment option for these patients.

Radiotherapy for pancreatic cancer remains challenging owing to its low radiosensitivity and the proximity of the pancreas to highly radiosensitive organs, such as the gastrointestinal (GI) tract. Recently, particle radiotherapy (PRT), such as proton or carbon ion therapy, has been increasingly used for the treatment of pancreatic cancer. PRT facilitates the selective irradiation of the tumor while reducing GI toxicity owing to the physical property of dose deposition, namely the Bragg peak [11–13]. Some studies have reported encouraging results of PRT for LAPC, including increased irradiation doses, improved local control, and prolonged OS [14–18]. However, patient backgrounds and treatment protocols varied among these studies; thus, it remains unclear which group of patients can achieve feasible outcomes with PRT.

We have previously reported the feasibility and efficacy of gemcitabine-concurrent proton radiotherapy (GPT) for LAPC in a phase I/II study [14]; subsequently, we have performed GPT on many patients with LAPC. This study identified its long-term outcomes in a large number of patients with LAPC treated with GPT and assessed the factors associated with long-term survival outcomes of GPT for LAPC.

## Methods

### Patients

The medical records of patients with LAPC (*n* = 306) treated with GPT between February 2009 and December 2019 at Hyogo Ion Beam Medical Center were retrospectively reviewed. Of the 306 patients, 44 who received GPT with protocol doses other than 67.5 Gy equivalents (GyE) were excluded: 18, 20, and 6 patients received 50 GyE in 25 fractions, 52 GyE in 26 fractions, and 70.2 GyE in 26 fractions, respectively. Additionally, 139 patients who had received prior treatment for the primary tumor were excluded. In total, 123 patients treated with GPT of 67.5 GyE in 25 fractions without prior treatment were enrolled in this study.

Abdominal contrast-enhanced computed tomography (CT), chest CT, and positron emission tomography with 18F-fluorodeoxyglucose (FDG-PET) were performed before GPT. The diagnosis of pancreatic cancer was confirmed histologically (*n* = 75) or clinically by diagnostic imaging, such as CT, magnetic resonance imaging, and/or ultrasonic endoscopy (*n* = 48). LAPC was defined as tumor contact with the superior mesenteric artery or celiac artery > 180° or unreconstructible superior mesenteric vein/portal vein due to tumor involvement or occlusion [19].

This study was approved by the Institutional Review Board of Hyogo Ion Beam Medical Center and conducted according to the ethical standards stated in the 1964 Declaration of Helsinki and its later amendments; the need for informed consent was waived owing to the retrospective nature of the study.

### Proton radiotherapy

The patients were treated with 150–210 MeV proton beams accelerated by a synchrotron following a linear accelerator (Hybrid Particle Therapy System; Hitachi Ltd., Tokyo, Japan), and a respiratory gating system was used to irradiate the beam during the exhalation phase. Patients were immobilized using a custom-made thermoplastic cast in the prone position, and the setup was performed daily before irradiation using bony landmarks and fiducial markers detained to a branch of the gastroduodenal and/or dorsal pancreatic artery by angiography. The treatment plans were developed using a 2-mm slice thickness CT-based three-dimensional treatment planning system (Mitsubishi Electric, Tokyo, Japan).

The gross tumor volume (GTV) was defined as the volume of the primary tumor and the apparent lymph nodes. The clinical target volume (CTV) comprised the addition of a 5-mm margin to the GTV, prophylactic irradiation regions containing the draining lymph nodes and para-aortic lymph nodes, and peripheral regions surrounding the celiac and superior mesenteric arteries. The planning target volume (PTV) was defined as the CTV with a 5-mm setup margin and a 1–5-mm respiratory gating margin, which was measured on CT images between inspiratory and expiratory phases. The total delivered doses were calculated according to the relative biological effectiveness (RBE), and the RBE value for the treatment beam was 1.1 [20]. Total doses of 67.5 GyE in 25 daily fractions were administered using the field-in-field technique [14]. In general, the stomach, small bowel including the duodenum, kidneys, and spinal cord were defined as organs at risk. The dose restrictions for the stomach, duodenum, and spinal cord were approximately 50, 50, and 45 GyE, respectively. Additionally, we planned the irradiated volumes of the stomach, duodenum, and kidneys to be as minimal as possible.

### Concurrent and adjuvant chemotherapy

Concurrent chemotherapy was provided with gemcitabine monotherapy. All patients were scheduled to receive intravenous infusions of gemcitabine (800 mg/m^2^) for the initial 3 weeks (days 1, 8, and 15) during 5 weeks of proton radiotherapy [14]. Gemcitabine was administered with an absolute granulocyte count of > 2000/mm^3^ and a platelet count of > 70,000/m^3^ on the scheduled day.

Following GPT, 106 patients received systemic chemotherapy. Three patients did not receive adjuvant chemotherapy: one rejected the therapy and two were excluded due to poor general condition. The details of the treatment were not available for the remaining 14 patients.

### Patient follow-up

Patients were followed up at our outpatient clinic every 3 months after GPT. Blood examinations and contrast-enhanced CT and/or FDG-PET were performed at every visit. Endoscopic examinations were performed to evaluate radiation-related gastroduodenal complications.

Local progression was defined as tumor progression inside the PTV and diagnosed comprehensively based on the following findings: enlarged tumor size, increased FDG accumulation, and sustained increase in tumor markers for at least 3 months without any distant metastases. OS was defined as the time interval between the initiation of GPT and death. Progression-free survival (PFS) was defined as the time interval between the initiation of GPT and the detection of local progression, occurrence of distant metastases, or death (all causes), whichever occurred first, and local PFS (LPFS) was defined as the time interval between the initiation of GPT and the detection of local progression or death (all causes), whichever occurred first. GPT toxicities were evaluated according to the National Cancer Institute Common Terminology Criteria for Adverse Events (version 4.0).

### Statistical analyses

Patient characteristics are described as medians (ranges), while survival times and rates are described as medians (95% confidence intervals [CIs]). To evaluate between-group differences, the χ^2^ test was used for categorical variables, and the Wilcoxon rank-sum test was used for continuous variables. The Kaplan–Meier method was used to estimate survival outcomes, such as OS, PFS, and LPFS, and the differences were evaluated using log-rank tests. Univariate and multivariate analyses with Cox proportional hazard models were performed to determine the factors associated with OS. Variables with a *P-*value < 0.1 in univariate analysis were included in the multivariate analysis, and those with a *P-*value < 0.05 were considered statistically significant. All statistical analyses were performed using the JMP 16 statistical package (SAS Institute, Cary, NC, USA).

## Results

### Patient characteristics

The baseline patient characteristics are summarized in Table 1. We examined 58 women (47%) and 65 men (53%), with a median age of 64 years (range: 38–84). Moreover, 59 (48%) and 64 (52%) patients had pancreatic head and pancreatic body-tail cancers, respectively. The median tumor size was 32 mm (11–68). On CT images obtained before irradiation, bile duct, duodenal, and anterior peripancreatic invasions were observed in 63 (51%), 64 (52%), and 104 (85%) patients, respectively. Posterior peripancreatic, venous, arterial, and extrapancreatic nerve plexus invasions were observed in all patients. All patients received GPT of 67.5 GyE in 25 fractions and completed the planned treatment. The median GTV volume and the volume ratio irradiated over 60 GyE (V60_GyE_) at the GTV were 44.0 cc (7.9–141.0) and 59.4% (6.2–99.7), respectively.

**Table 1:** Patient characteristics

Variables	Number of patients (n = 123)
Median age, years (range)	64 (38–84)
Gender, n (%)	
Male	65 (53)
Female	58 (47)
ECOG-PS, n (%)	
0	94 (76)
1	27 (22)
2	2 (2)
Tumor location, n (%)	
Head	59 (48)
Body-Tail	64 (52)
Median CEA, ng/mL (range)	3.3 (0.7–70.1)
Median CA19-9, U/mL (range)	366.8 (0.1–27,600)
Adjuvant therapy, n (%)	
Yes	106 (87)
No	3 (2)
Unknown	14 (11)
Pathological diagnosis, n (%)	
Yes	75 (61)
No	48 (39)
Median tumor diameter, mm (range)	32 (11–68)
Lymph node metastasis, n (%)	
Positive	41 (33)
Negative	82 (67)
Bile duct invasion, n (%)	
Positive	63 (51)
Negative	60 (49)
Duodenal invasion, n (%)	
Positive	64 (52)
Negative	59 (48)
Anterior peripancreatic invasion, n (%)	
Positive	104 (85)
Negative	19 (15)
Posterior peripancreatic invasion, n (%)	
Positive	123 (100)
Negative	0 (0)
Venous invasion, n (%)	
Positive	123 (100)
Negative	0 (0)
Arterial invasion, n (%)	
Positive	123 (100)
Negative	0 (0)
Extrapancreatic nerve plexus invasion, n (%)	
Positive	123 (100)
Negative	0 (0)
Median GTV volume, cc (range)	44.0 (7.9–141.0)
Median CTV volume, cc (range)	186.5 (85.0–420.6)
Median GTV V60_GyE_, % (range)	59.4 (6.2–99.7)
Median CTV V60_GyE_, % (range)	59.3(25.6–86.7)

*ECOG-PS* Eastern Cooperative Oncology Group-Performance Status; *CEA* carcinoembryonic antigen; *CA19-9* carbohydrate antigen 19–9; *GTV* gross tumor volume; *CTV* clinical target volume; *GyE* Gy equivalents; *V60*_*GyE*_ the volume ratio irradiated over 60 GyE

### Patient survival

**Fig. 1:**
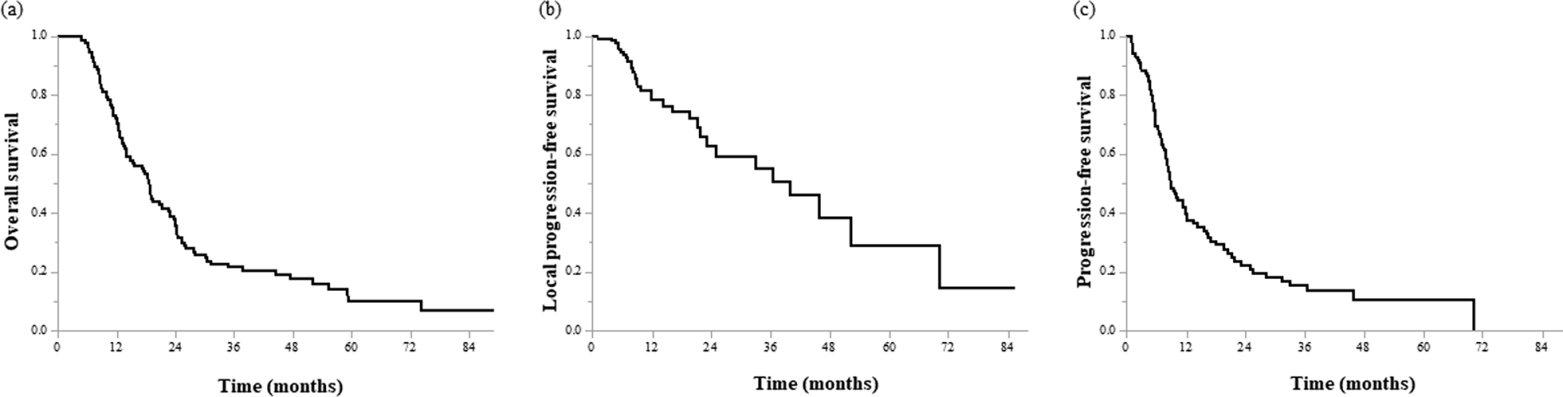
Survival curves for all patients. **a** Overall survival, **b** local progression-free survival, and **c** progression-free survival

The median follow-up time was 15.2 months (4.4–89.2). The median overall survival (mOS) was 18.7 months (95% CI: 14.7–22.9). The 1- and 2-year OS rates were 70.4% (62.6–79.2) and 35.7% (27.7–46.0), respectively (Fig. 1a). The 1- and 2-year LPFS rates were 78.2% (69.7–87.6) and 59.0% (46.2–75.4), respectively (Fig. 1b). The 1- and 2-year PFS rates were 38.6% (30.2–49.3) and 20.8% (13.7–31.5), respectively (Fig. 1c). Local progression developed in 18 (15%) patients, whereas distant metastases developed in 58 (47%) patients within 1 year. In total, 74 (60%) patients experienced distant metastases, including 36 (29%) with liver metastasis, 30 (24%) with peritoneal dissemination, and 15 (12%) with lung metastasis.

### Toxicity

Acute toxicities of grades 3 and 4 were observed in 52 (42%) and 3 patients (2%), respectively. All grade 3 and 4 toxicities were hematologic, including leukopenia, neutropenia, and thrombocytopenia in 55 (45%), 28 (23%), and 4 patients (3%), respectively. There were no grade 5 acute toxicities or treatment-related deaths. Regarding late toxicities, 6 (5%) patients experienced grade 3 toxicities comprising bile duct stenosis, duodenal stenosis, and gastric hemorrhage in 1 (1%), 1 (1%), and 4 (3%) patients, respectively. Two (2%) patients experienced grade 4 gastric hemorrhage. Three (2%) patients experienced grade 5 toxicities, including duodenal perforation and bile duct perforation with a metallic biliary stent in 1 (1%) and 2 (2%) patients, respectively.

### Prognostic factors

Univariate analysis revealed that LAPCs at the pancreatic body-tail or those without bile duct, duodenal, or anterior peripancreatic invasions were associated with longer OS than those at the pancreatic head or those with bile duct, duodenal, or anterior peripancreatic invasions (Table 2). In multivariate analysis, LAPCs at the pancreatic body-tail (hazard ratio [HR]: 0.12, 95% CI: 0.02–0.91, *P* = 0.040) and those without anterior peripancreatic invasion (HR: 0.46, 95% CI: 0.25–0.86, *P* = 0.015) were significantly associated with longer OS (Table 2).

**Table 2:** Univariate and multivariate analyses of prognostic factors for overall survival

		Univariate		Multivariate	
Variables	n (%)	HR (95% CI)	*P* value	HR (95% CI)	*P* value
Age ≧ 65 years	57 (46)	1.47 (0.97–2.22)	0.069	1.52 (0.99–2.33)	0.053
Gender, Male	65 (53)	0.82 (0.55–1.24)	0.351		
ECOG-PS, 0	94 (76)	0.93 (0.59–1.47)	0.744		
Tumor location, Body-tail	64 (52)	0.50 (0.33–0.76)	**0.001**	0.12 (0.02–0.91)	**0.040**
CEA ≧ 5 ng/mL	34 (28)	1.27 (0.82–1.97)	0.291		
CA19-9 ≧ 37 U/mL	104 (85)	0.98 (0.55–1.74)	0.940		
Pathological diagnosis, Yes	75 (61)	0.89 (0.59–1.35)	0.589		
Lymph node metastasis, Negative	82 (67)	1.04 (0.68–1.60)	0.846		
Bile duct invasion, Negative	60 (49)	0.61 (0.40–0.92)	**0.018**	7.22 (0.76–68.4)	0.085
Duodenal invasion, Negative	59 (48)	0.57 (0.38–0.87)	**0.009**	0.55 (0.19–1.63)	0.283
Anterior peripancreatic invasion, Negative	19 (15)	0.50 (0.27–0.92)	**0.027**	0.46 (0.25–0.86)	**0.015**
GTV V60_GyE_ ≧ 60%	59 (48)	0.77 (0.51–1.16)	0.208		

*HR* hazard ratio; *CI* confidence interval; *ECOG-PS* Eastern Cooperative Oncology Group-Performance Status; *CEA* carcinoembryonic antigen; *CA19-9* carbohydrate antigen 19–9; *GTV* gross tumor volume; *GyE* Gy equivalents; *V60*_*GyE*_ the volume ratio irradiated over 60 GyE

Significant *P* values (< 0.05) are in bold

**Fig. 2:**
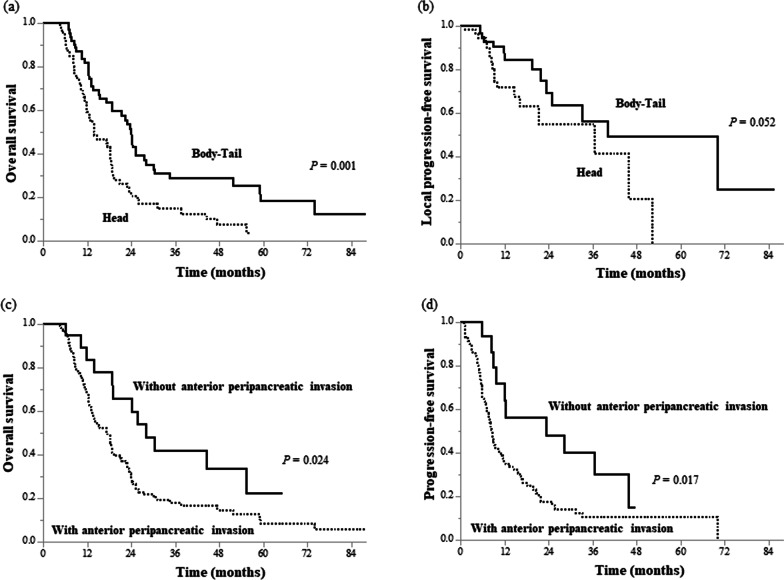
Survival curves for subsets of patients. Overall survival (**a**) and local progression-free survival (**b**) curves of patients with locally advanced pancreatic cancer (LAPC) at the pancreatic head and body-tail. Overall survival (**c**) and progression-free survival (**d**) curves of patients with LAPC with and without anterior peripancreatic invasion

LAPCs at the pancreatic body-tail had a significantly longer mOS than those at the pancreatic head (24.1 [18.8–30.2] vs. 14.0 [12.1–18.9] months, *P* = 0.001; Fig. 2a). The median LPFS times of LAPCs at the pancreatic body-tail and pancreatic head were 40.1 (25.0–NA) and 36.6 (16.1–NA) months, respectively (*P* = 0.052, Fig. 2b). There was no significant difference between the PFS of the two groups. Table 3 presents the dose intensity based on the dose volume histogram differences between the groups. GTV V60_GyE_ was significantly higher in LAPCs at the pancreatic body-tail than in those at the pancreatic head (66.8 vs. 50.0%, *P* < 0.001).

**Table 3:** Dose intensity based on the dose volume histogram for tumor location

	Head	Body-tail	
Parameter	Median value (range)	Median value (range)	*P* value
GTV			
Volume, cc	39.2 (7.9–88.9)	49.2 (9.1–141.0)	**0.038**
V60_GyE_, %	50.0 (6.2–98.7)	66.8 (25.2–99.7)	** < 0.001**
Dmax, GyE	68.7 (66.8–72.0)	68.8 (66.8–71.1)	0.406
Dmean, GyE	58.8 (48.8–66.4)	61.4 (50.7–67.3)	** < 0.001**
Dmin, GyE	45.0 (40.0–55.6)	45.2 (36.5–58.3)	0.752
CTV			
Volume, cc	175.5 (89.7–417.0)	196.3 (85.0–420.6)	0.434
V60_GyE_, %	58.7 (25.6–82.7)	61.2 (37.7–86.7)	0.223
Dmax, GyE	69.7 (67.7–72.7)	69.7 (68.0–71.7)	0.285
Dmean, GyE	60.3 (52.1–64.7)	60.6 (54.8–65.5)	0.207
Dmin, GyE	44.3 (31.6–47.6)	44.4 (38.2–50.2)	0.697
PTV			
Volume, cc	340.1 (184.3–728.6)	364.3 (175.7–696.5)	0.535
V60_GyE_, %	47.9 (20.3–69.1)	48.4 (27.4–75.3)	0.335
Dmax, GyE	69.9 (67.7–72.7)	69.8 (68.0–72.2)	0.192
Dmean, GyE	57.6 (48.8–62.3)	57.7 (52.3–63.4)	0.222
Dmin, GyE	37.2 (23.0–41.4)	37.4 (28.5–45.1)	0.208

*GTV* gross tumor volume; *CTV* clinical target volume; *PTV* planning target volume; *GyE* Gy equivalents; *V60*_*GyE*_ the volume ratio irradiated over 60 GyE; *Dmax* the maximum dose of the target volume; *Dmean* the average dose of the target volume; *Dmin* the minimum dose of the target volume

Significant *P* values (< 0.05) are in bold

**Fig. 3:**
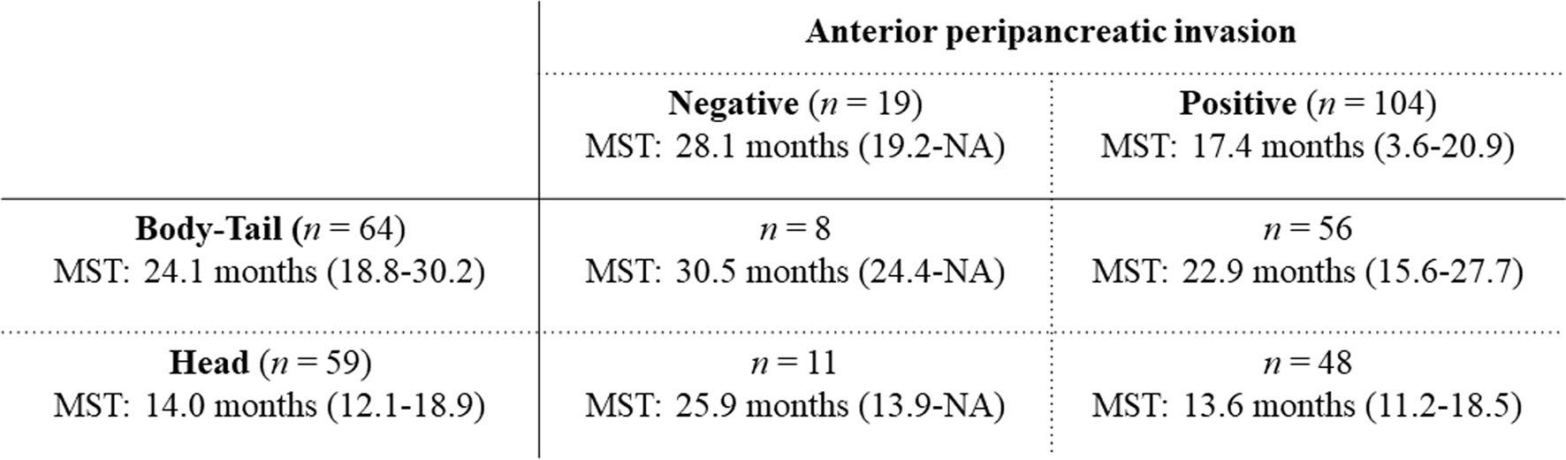
Median survival times of patients with locally advanced pancreatic cancers at the pancreatic head and body-tail in combination with and without anterior peripancreatic invasion

**Fig. 4:**
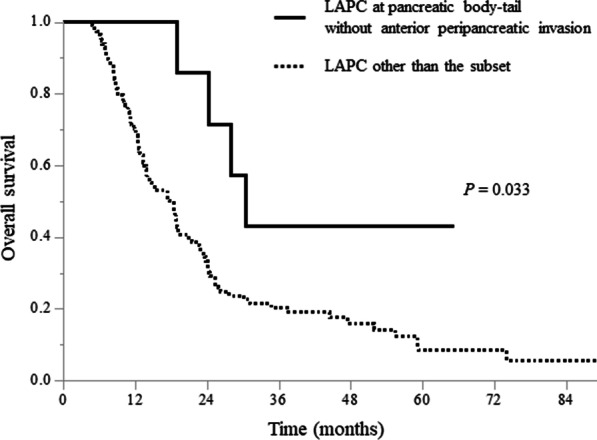
Comparison of overall survival curves between patients with locally advanced pancreatic cancer (LAPC) at the pancreatic body-tail without anterior peripancreatic invasion and those with LAPC other than the subset

LAPCs without anterior peripancreatic invasion also had significantly longer mOS than those with anterior peripancreatic invasion (28.1 [19.2–NA] vs. 17.4 [3.6–20.9] months, *P* = 0.024; Fig. 2c). Although there was no significant difference in the LPFS, the 1- and 2-year PFS rates were 55.8% (34.6–90.1) and 39.9% (20.4–77.9) in LAPCs without anterior peripancreatic invasion, respectively, which were significantly higher than those in LAPCs with anterior peripancreatic invasion (34.6% [26.0–46.1] and 15.8% [9.2–27.4], respectively, *P* = 0.017; Fig. 2d). LAPCs with anterior peripancreatic invasion had more peritoneal recurrence within 6 months after GTP than those without anterior peripancreatic invasion (*P* = 0.039, Table 4). The mOSs of the LAPCs at the pancreatic head and body-tail in combination with and without anterior peripancreatic invasion are summarized in Fig. 3. LAPCs at the pancreatic body-tail without anterior peripancreatic invasion were associated with significantly longer OS than those at the pancreatic head with and without anterior peripancreatic invasion or those at the pancreatic body-tail with anterior peripancreatic invasion (30.5 [24.4–NA] vs. 17.7 [13.9–21.4] months, *P* = 0.033, Fig. 4).

**Table 4:** Recurrence pattern according to each clinical feature

		Anterior peripancreatic invasion			Tumor location		
Variables	n (%)	Positive (n = 104)	Negative (n = 19)	*P* value	Head (n = 59)	Body-Tail (n = 64)	*P* value
Local progression, n (%)				0.555			0.275
Yes	32 (26)	26 (25)	6 (32)		18 (31)	14 (22)	
No	91 (74)	78 (75)	13 (68)		41 (69)	50 (78)	
Local progression within 6 months, n (%)				0.119			0.780
Yes	7 (6)	7 (7)	0 (0)		3 (5)	4 (6)	
No	116 (94)	97 (93)	19 (100)		56 (95)	60 (94)	
Local progression within 1 year, n (%)				0.568			0.084
Yes	18 (15)	16 (15)	2 (11)		12 (20)	6 (9)	
No	105 (85)	88 (859	17 (89)		47 (80)	58 (91)	
Distant metastasis, n (%)				**0.026**			0.853
Yes	74 (60)	67 (64)	7 (37)		36 (61)	38 (59)	
No	49 (40)	37 (36)	12 (63)		23 (39)	26 (41)	
Liver metastasis within 6 months, n (%)				0.066			0.988
Yes	23 (19)	22 (21)	1 (5)		11 (19)	12 (19)	
No	100 (81)	82 (79)	18 (95)		48 (81)	52 (81)	
Liver metastasis within 1 year, n (%)				0.099			0.870
Yes	30 (24)	28 (27)	2 (11)		14 (24)	16 (25)	
No	99 (76)	76 (73)	17 (89)		45 (76)	48 (75)	
Peritoneal dissemination within 6 months, n (%)				**0.039**			0.882
Yes	12 (10)	12 (12)	0 (0)		6 (10)	6 (9)	
No	111 (90)	92 (88)	19 (100)		53 (90)	58 (91)	
Peritoneal dissemination within 1 year, n (%)				0.584			0.656
Yes	25 (20)	22 (21)	3 (16)		11 (19)	14 (22)	
No	98 (80)	82 (79)	16 (84)		48 (81)	50 (78)	

Significant *P* values (< 0.05) are in bold

## Discussion

Controversies surrounding the optimal treatment strategy for LAPC exist. Many physicians consider that systemic chemotherapy is the sole effective treatment for LAPC; however, a considerable proportion of patients do not develop distant metastases, and local tumor progression is the only cause of death [9, 10]. GPT is an attractive treatment for LAPC without distant metastases because of its high potential for local tumor control [14]. In this study, we successfully reported high LPFS rates (1- and 2-year LPFS rates of 78.2% and 59.0%, respectively) in patients with LAPC treated with GPT of 67.5 GyE; the mOS was 18.7 months and the 1- and 2-year OS rates were 70.4% and 35.7%, respectively. These values are comparable to those of intensive chemotherapies, such as FOLFIRINOX and GnP (mOSs, 24.2 and 18.8 months, respectively) [5, 6], photon radiotherapy with 50–54 GyE (mOS, 9.6–17.6 months; 2-year OS rate, 10.5–28%) [21–25], proton radiotherapy with 50–67.5 GyE (mOS, 18.4–25.6 months; 2-year OS rate, 31–53%) [15–18], or intensity-modulated radiation therapy (IMRT) with 50.4–70.4 GyE (mOS, 17.8–21.4 months) [26, 27]. PRT reportedly allows sparing of adjacent normal tissues and offers incrementally better dosimetric coverage by the Bragg peak compared to IMRT [11, 12]. In addition, a potential superior anti-tumor effect of protons compared to photons has been indicated [28]. Long mOS was achieved in patients with LAPC at the pancreatic body-tail and those with LAPC without anterior peripancreatic invasion (24.1 and 28.1 months, respectively), suggesting that GPT is a promising treatment option, especially for this subset of patients.

LAPCs at the pancreatic body-tail had a significantly higher GTV V60GyE than those at the pancreatic head (66.8 vs. 50.0%), as the irradiation doses were consistently restricted by the adjacent second and third portions of the duodenum in LAPCs at the pancreatic head. Similar to the well-known consequence that dose escalation improves OS and LPFS [17, 26, 29], OS and LPFS were better in patients with LAPC at the pancreatic body-tail than in those at the pancreatic head. However, even GTV V60GyE of LAPCs at the pancreatic body-tail was insufficient for curative irradiation due to the restriction of irradiation doses to the adjacent GI tract. To further increase irradiation doses to the entire tumor volume, we developed a new conceptual approach called the space-making particle therapy, wherein we performed surgical spacer placement and subsequent PRT. Significant dose escalation by space-making particle therapy has been reported in LAPCs (mean GTV V60GyE, 66.4 to 84.7%) [30], and the effect of this new technique on OS and LPFS is anticipated to be further elucidated in future studies.

LAPC often undergoes distant metastasis, and the role of radiotherapy as systemic therapy may be limited. Our study demonstrated that GPT contributes to a high LPFS, albeit an insufficient PFS, suggesting that GPT provides feasible local tumor control but does not contribute to the control of distant metastasis. Therefore, an optimal patient selection with a low risk of distant metastasis is required to improve the survival outcomes of GPT. Our results also indicate that anterior peripancreatic invasion is a risk factor for peritoneal dissemination within 6 months after GTP. Furthermore, approximately 10–20% of patients with radiographically diagnosed LAPC have occult peritoneal dissemination [31, 32]. Thus, exploratory laparoscopy before GPT may be recommended for LAPC with anterior peripancreatic invasion to exclude radiologically negative peritoneal dissemination cases. Other studies have reported that induction chemotherapy before chemoradiotherapy is also useful in excluding LAPC with occult metastases [33–35]. A tailored treatment strategy based on the condition of each patient could achieve better survival outcomes of LAPC.

In this study, the frequency of acute toxicities of grade ≥ 3 (45%) was comparable to that in a previous report on chemotherapy concurrent with PRT (45%) [19]. Although a direct comparison is difficult, GPT is a relatively safer treatment than FOLFIRINOX (60%) and GnP (80%) [5, 6]. Moreover, GPT may be administered to patients who have difficulty receiving intensive chemotherapy owing to serious adverse events. We observed that all acute toxicities of grade ≥ 3 were hematologic and there was no difference in the frequency of acute toxicities between LAPCs at the pancreatic head and body-tail. On the other hand, there were more clinically significant radiation-induced late toxicities of grade ≥ 3 in relation to the bile duct and GI tract in LAPCs at the pancreatic head than in those at the body-tail. This was partially attributed to the proximity of the LAPCs at the pancreatic head to the bile duct and GI tract. Late toxicities of grade ≥ 3 for the bile duct and GI tract should not be neglected. Based on our clinical experience, bile duct perforation is more likely to occur in patients with a metallic biliary stent, and we invariably use a plastic biliary stent. Additionally, we religiously used a proton pump inhibitor and mucosal protective agent to prevent peptic ulcer diseases.

This study had some limitations. It was a small, retrospective, non-randomized study at a single institution, and our patient population might have been biased toward favoring the effectiveness of GPT. Moreover, adjuvant treatment after GPT was performed at other institutions, and there were insufficient data on the treatment and clinical course after GPT. A multi-institutional prospective study would reduce the possibility of bias and provide a more conclusive result on the factors associated with long-term survival outcomes of GPT for LAPC.

## Conclusions

Patients with LAPC at the pancreatic body-tail and those with LAPC without anterior peripancreatic invasion showed favorable OS after GPT. With appropriate patient selection based on the accessibility of irradiation and the risk of metastases, GPT would aid in the achievement of better survival outcomes, thus showing a promising potential as a treatment option for LAPC.

## Data Availability

The datasets used and analyzed during the current study are available from the corresponding author on reasonable request.

## Abbreviations

CI: Confidence interval
CT: Computed tomography
CTV: Clinical target volume
FDG-PET: Positron emission tomography with 18F-fluorodeoxyglucose
FOLFIRINOX: 5-Fluorouracil, leucovorin, irinotecan, and oxaliplatin
GI: Gastrointestinal
GnP: Gemcitabine plus nab-paclitaxel
GPT: Gemcitabine-concurrent proton radiotherapy
GTV: Gross tumor volume
GyE: Gy equivalents
HR: Hazard ratio
IMRT: Intensity-modulated radiation therapy
LAPC: Locally advanced pancreatic cancer
LPFS: Local progression-free survival
mOS: Median overall survival
NA: Not available
OS: Overall survival
PFS: Progression-free survival
PRT: Particle radiotherapy
PTV: Planning target volume
RBE: Relative biological effectiveness
V60_GyE_: The volume ratio irradiated over 60 GyE
